# Normal Pregnancy Is Associated with Changes in Central Hemodynamics and Enhanced Recruitable, but Not Resting, Endothelial Function

**DOI:** 10.1155/2015/250951

**Published:** 2015-09-02

**Authors:** Juan Torrado, Yanina Zócalo, Ignacio Farro, Federico Farro, Claudio Sosa, Santiago Scasso, Justo Alonso, Daniel Bia

**Affiliations:** ^1^Centro Universitario de Investigación, Innovación y Diagnóstico Arterial (CUiiDARTE), Physiology Department, Faculty of Medicine, Republic University, General Flores 2125, 11800 Montevideo, Uruguay; ^2^Department of Obstetrics and Gynecology “C”, Pereira-Rossell Hospital, Faculty of Medicine, Republic University, Br. Artigas 1550, 11600 Montevideo, Uruguay

## Abstract

*Introduction.* Flow-mediated dilation (FMD), low flow-mediated constriction (L-FMC), and reactive hyperemia-related changes in carotid-to-radial pulse wave velocity (ΔPWVcr%) could offer complementary information about both “recruitability” and “resting” endothelial function (EF). Carotid-to-femoral pulse wave velocity (PWVcf) and pulse wave analysis-derived parameters (i.e., AIx@75) are the gold standard methods for noninvasive evaluation of aortic stiffness and central hemodynamics. If healthy pregnancy is associated with both changes in resting and recruitable EF, as well as in several arterial parameters, it remains unknown and/or controversial. *Objectives.* To simultaneously and noninvasively assess in healthy pregnant (HP) and nonpregnant (NP) women central parameters in conjunction with “basal and recruitable” EF, employing new complementary approaches. *Methods.* HP (*n* = 11, 34.2 ± 3.3 weeks of gestation) and age- and cardiovascular risk factors-matched NP (*n* = 22) were included. Aortic blood pressure (BP), AIx@75, PWVcf, common carotid stiffness, and intima-media thickness, as well as FMD, L-FMC, and ΔPWVcr %, were measured. *Results.* Aortic BP, stiffness, and AIx@75 were reduced in HP. ΔPWVcr% and FMD were enhanced in HP in comparison to NP. No differences were found in L-FMC between groups. *Conclusion.* HP is associated with reduced aortic stiffness, central BP, wave reflections, and enhanced recruitable, but not resting, EF.

## 1. Introduction

Arterial structure and function can now be simple and noninvasively assessed by different accurate methods, which have been extensively used in patients with cardiovascular risk factors. In this context, Celermajer et al.'s technique, commonly known as* flow-mediated dilation* (FMD), which utilizes the vasoreactivity test (VRT), has stood the test of time and remains the most popular method to assess endothelial function [1, 2]. The VRT consists in positioning a pneumatic cuff around the forearm and provoking an arterial occlusion for five minutes (i.e., transient ischemia). This maneuver elicits an increase in blood flow in the brachial artery once the cuff is deflated (i.e., reactive hyperemia,* RH*), which subsequently stimulates endothelium to release several vasoactive biochemical factors (i.e., nitric oxide). Finally, locally produced factors result in a dilation of the brachial artery (measured by B-Mode ultrasound) [2] and a reduction in arterial stiffness (changes in pulse wave velocity [PWV] assessed by mechanotransducers [3]). The VRT has also been also applied in healthy pregnancy and in pregnancy-related diseases [4, 5]. Independently of the setting, the magnitude of the arterial dilation is used as an indicator of endothelial function and healthy pregnant women show an enhanced vascular response evaluated by this method compared with healthy nonpregnant women [6, 7]. However, whereas FMD provides information about the “recruitability” of endothelial function (i.e., its responsiveness to a specific stimulus), it does not provide information concerning basal/tonic endothelial function (i.e., release of endothelial autacoids before FMD measures are initiated) [8]. In this context, Gori et al. described a novel index for assessing the response of the artery to low flow, which utilizes data obtained from the cuff occlusion period of an FMD scan [9]. Synonymous to FMD, the vasoconstriction observed under conditions of reduced blood flow has been named low-flow-mediated vasoconstriction (L-FMC) [9]. Inclusion of L-FMC data to traditional measurement of FMD could provide additional and/or complementary information, which, they propose, may improve the detection of patients with cardiovascular disease and profile the vascular response to exercise among healthy volunteers [10]. Whether healthy pregnancy is associated with changes in L-FMC remains to be established. In this context, it is also unknown if the integration of L-FMC into traditional FMD studies will be able to provide additional/complementary information among pregnant women.

In addition, changes in carotid-to-radial PWV (PWVcr) due to the same maneuver have been proposed as an alternative tool for the evaluation of recruitable endothelial function [3, 11]. PWV is recognized as the “gold standard” parameter for the evaluation of regional arterial stiffness and has had a wide biomedical application [12, 13]. A reduction in PWVcr values has been reported in response to VRT in healthy young adults [3] whereas a blunted reduction has been reported in pathophysiological circumstances, such as hypertension [14] and congestive heart failure [11]. A preliminary report suggested that changes in PWVcr due to VRT offer additional information of endothelial dynamics and thus could have a potential role in the assessment of endothelial function during pregnancy with a potential clinical application in predicting pregnancy-induced hypertension and preeclampsia [15].

Finally, there is still a lack of knowledge about the expected changes for several structural and functional parameters widely used in noninvasive arterial studies. In this regard, if pregnancy is associated with changes in central aortic pressure, wave reflections levels and/or elastic and muscular arteries stiffness remains to be determined. Changes in arterial structure and function of both maternal muscular and elastic arteries could be of value in accommodating pregnancy-induced increasing cardiac output and blood volume for a correct maternal-fetal hemodynamic interaction [16, 17]. Therefore, we here hypothesize that normal pregnancy, in contraposition to the nonpregnant status and particularly during the third trimester stage in which profound cardiovascular changes are expected, will evidence notable vascular adaptations (i.e., reduction in wave reflections levels and arterial stiffness), features capable of being assessed and quantified by using promising and complementary noninvasive arterial parameters.

In this context, the work aims were firstly to determine and analyze “basal and recruitable” endothelial function through the measurement of brachial artery FMD and L-FMC and PWVcr RH-related changes in a group of healthy nonpregnant and pregnant women and, secondly, to determine noninvasively central and peripheral arterial parameters, by using validated and gold standard techniques.

## 2. Methods

### 2.1. Subjects, Demographic Characteristics, and Laboratory Samples

This was an analytic observational case-control study involving 11 healthy pregnant (HP) and 22 healthy nonpregnant women (NP). The HP women were recruited from the routine antenatal clinic where they were asked and agreed to participate in the study (convenience sampling). NP group was obtained from our database (CUiiDARTE Project and Centre, Republic University) once HP women were matched based on age, height, and cardiovascular risk factors [18–20]. Baseline demographic and anthropometric data were obtained by an obstetrician/physician during a clinical interview and exam, and laboratory samples were extracted prior to the examination. They were all healthy (with the exception of dyslipidemia in some of them) and without family history of premature cardiovascular disease. All HP women had uncomplicated pregnancies before and during the study. None of them received any vasoactive drugs.

Exclusion criteria for HP and NP included previous history of pregnancy-induced hypertension (including preeclampsia), gestational diabetes, or current chronic hypertension and/or diabetes mellitus. Significant unexplained proteinuria (>300 mg total protein in a 24 h urine collection) developing in obstetric controls was also an exclusion criterion for HP. The definitions for pathologies used for exclusion criteria took into account the one recommended by the National Institute for Health and Clinical Excellence guidelines [21, 22].

Participants were asked to abstain from physical activity, tobacco products, and vitamin supplementation for at least 4 hours prior to the examination. The study protocol was approved by the Ethics Research Committee of the Republic University (Uruguay) and all participants gave written informed consent.

### 2.2. Baseline Noninvasive Arterial Evaluation

Subjects were instructed to lie in a left lateral position (particularly for HP, to avoid vena cava compression by the uterus) in a temperature-controlled (21°–23°C) room, for at least 15 minutes, in order to establish stable hemodynamic conditions. Heart rate (HR) and right brachial (peripheral) systolic and diastolic blood pressure (pSBP and pDBP, resp.) were measured using an oscillometric device (Omron HEM-433INT Oscillometric System; Omron Healthcare Inc., Illinois, USA) at 5–8-minute intervals during the whole procedure. Mean blood pressure (MBP) was determined using classic empirical formula currently used at the peripheral level as pDBP plus one-third times of peripheral pulse pressure (pPP = pSBP − pDBP).

#### 2.2.1. Carotid-to-Femoral Pulse Wave Velocity and Pulse Wave Analysis

**Figure 1:**
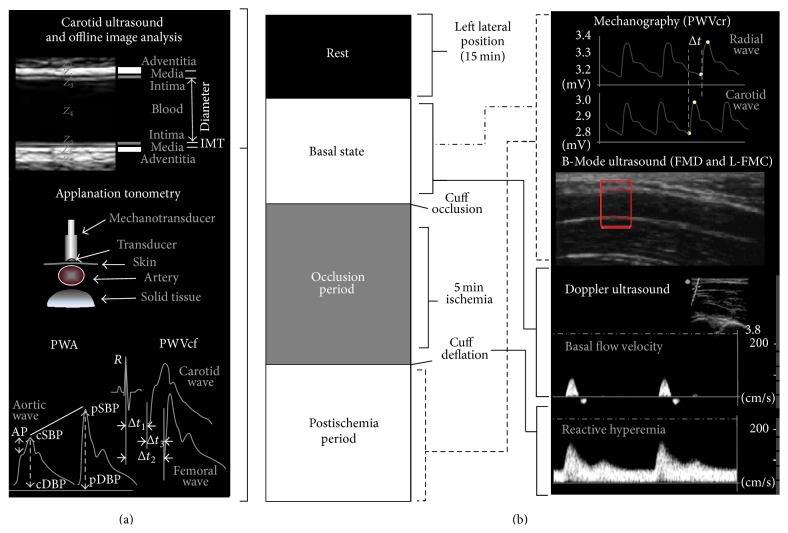
(a) Schema of the instrumental approach employed to acquire noninvasively arterial parameters in basal conditions. Employed techniques: carotid-to-femoral PWV and PWA (applanation tonometry) and carotid arterial diameter and CIMT (B-Mode ultrasound). (b) Representative diagram of the study protocol of Vasoreactivity Test (VRT) applied to evaluate changes in carotid-to-radial PWV and brachial arterial diameter. *Z*: acoustic impedance; CIMT: carotid intima-media thickness; PWA: pulse wave analysis; PWVcf: carotid-to-femoral pulse wave velocity; Δ*t*_1_, Δ*t*_2_: time delay between *R* wave from ECG and central (carotid) foot wave and peripheral (femoral or radial) foot wave, respectively; Δ*t*_3_: time delay between carotid foot wave and radial foot wave; AP: augmentation pressure; cSBP and pSBP: central and peripheral systolic blood pressure, respectively; cDBP and pDBP: central and peripheral diastolic blood pressure, respectively; PWVcr: carotid-to-radial pulse wave velocity; FMD: flow-mediated dilation; L-FMC: low flow-mediated constriction.

The carotid-femoral pulse-wave velocity (PWVcf) was measured to analyze aortic regional stiffness. To this end, carotid and femoral artery waveforms were consecutively obtained with a high-fidelity applanation tonometer from the carotid and femoral regions simultaneously with continuous ECG monitoring (SphygmoCor 7.01, AtCor Medical, Sydney, Australia) (Figure 1). Then, carotid-femoral propagation time (Δ*t*_3_) was determined by subtracting the time delay between the peak of *R* wave of the ECG recording to femoral foot of the pressure waveform (Δ*t*_2_) of the corresponding cardiac cycle and the time delay between the peak of *R* wave to carotid foot of the pressure waveform (Δ*t*_1_). The algorithm utilized to detect the so-called “foot of the wave” was the* intersecting tangents*, explained elsewhere [23]. Straight distance between the recording sites (carotid-to-femoral distance [C-F  Δ*x*]) was then carefully measured using tape on the body surface to reduce the influence of altered body contour in pregnancy. Finally, PWVcf was automatically calculated as the quotient between C-F Δ*x* and Δ*t*_3_ (Figure 1). The reported value of PWVcf for a subject was always the average of at least eight consecutive beats.

Pulse wave analysis (PWA) was used to assess central hemodynamics as well as systemic arterial stiffness and wave reflections. For this porpoise, mean radial artery waveform was obtained (through the acquisition of many cycles) with the applanation tonometer from the wrist, and a corresponding mean ascending aortic pressure waveform was generated with a validated generalized transfer function using the same mentioned customized software (SphygmoCor 7.01, AtCor Medical, Sydney, Australia) [24]. The radial pulse waveform was then calibrated using the diastolic and mean arterial pressure obtained at the brachial artery [12]. Central systolic, diastolic, and pulse pressure (cSBP, cDBP, and cPP, resp.), heart rate corrected central augmentation index (*AIx@75*, adjusted to a rate of 75 beats/minute), and amplification ratio (pPP/cPP) were determined with the integrated software.

#### 2.2.2. Carotid Artery Studies

Ultrasound assessment of carotid arteries was based on the techniques and recommendations described in international consensus [25]. High-resolution B-Mode ultrasound images of both (right and left) common carotid arteries (CCA) were obtained using a linear-array transducer connected to a portable Ultrasound System (Probe L38e, 5–10 MHz, SonoSite, MicroMaxx, SonoSite Inc., 21919 30th Drive SE, Bothell, WA 98021, USA). Measurements (still images and video clips/cine loops) were digitally stored for offline analysis (Figure 1). Near and far walls were analyzed and images were obtained from anterior, lateral, and posterior angles. At first, a carotid plaque screening was performed, for which the definition used was a focal wall thickening at least 50% greater than that of the surrounding vessel, a thickening that protrudes into the lumen 0.5 mm or as a region with carotid intima-media thickness (CIMT) greater than 1.5 mm [25]. Then, longitudinal views of the CCAs were acquired and a video (cine loop) of at least 30 seconds was recorded and stored. The CIMT and beat-to-beat diameter waveforms were obtained and analyzed offline using a step-by-step border detection algorithm (based in changes in acoustic impedance [*Z*]), applied to each digitized image (Hemodyn-4M software, Buenos Aires, Argentina). A region 1.0 cm proximal to the carotid bulb was identified, and the far wall CIMT was determined as the distance between the lumen-intima and the media-adventitia interfaces (Figure 1). The software performs multiple automated or semiautomated measurements along the centimeter and averages them, increasing the accuracy of the measures.

The instantaneous mean diameter (from the leading edge of the near wall intima-media interface to the intima-media interface of the far wall) waveform was obtained during pulsation in order to obtain diastolic and systolic diameter. Then, the pressure strain or Peterson's elastic modulus (*E*_*P*_) was calculated relating these measures with central blood pressure as follows [12]:

(1) $$\begin{array}{c} E_{P}=\frac{\left(\text{cSBP}-\text{cDBP}\right)}{\left(\text{SD}-\text{DD}\right)/\text{DD}}, \end{array}$$

where cSBP, cDBP, SD, and DD are central systolic and diastolic blood pressure and carotid systolic and diastolic diameter, respectively (Figure 1). *E*_*P*_ measures the ability of the arteries to change its dimensions in response to the pulse pressure caused by cardiac pulsatile ejection (pressure change required for (theoretic) 100% increase in diameter) [12, 26, 27].

### 2.3. Vascular Reactivity: Resting and Recruitable Endothelial Function

Once baseline noninvasive arterial evaluation was carried out, we utilized the theoretical basis, general protocol, and methodological aspects of the VRT recommended by the guidelines for the ultrasound assessment of endothelial-dependent flow-mediated vasodilation of the brachial artery [2, 28]. For this purpose, participants were submitted to five minutes of ischemia by occluding left radial and cubital arteries using a pneumatic cuff placed around the left forearm (just below the elbow to at least 50 mm Hg above pSBP) and several parameters of vascular reactivity were measured before, during, and after ischemia (Figure 1). The parameters used for the evaluation of endothelial function are listed below.

#### 2.3.1. L-FMC, FMD, and Shear Rate

Taking into account “gold standard” accepted methodology for the evaluation of endothelial function “recruitability” and simultaneously PWVcr measurement (see later), left brachial artery was visualized longitudinally above the antecubital crease using the same high resolution B-Mode ultrasound device mentioned earlier (Sonosite; MicroMaxx; USA) (Figure 1). Similarly, video sequences were recorded at rest (1 minute), during forearm occlusion (5 minutes), and after cuff deflation (4 minutes). Subsequently and similarly to the processing of carotid images, recordings were analyzed offline using same automated step-by-step algorithm applied to each digitalized image that allows for the brachial diameter waveform obtainment and L-FMC and FMD calculation [29].

L-FMC was quantified as the percentage of change in brachial artery diastolic diameter (DD), considering the basal levels and the DD before cuff deflation:

(2) $$\begin{array}{c} \text{L-FMC}\%=\frac{{\text{DD}}_{\text{before cuff deflation}}-{\text{DD}}_{\text{baseline}}}{{\text{DD}}_{\text{baseline}}}\times 100. \end{array}$$

FMD was quantified as the percentage of change in brachial DD, considering the basal levels and the maximal diastolic diameter after cuff deflation:

(3) $$\begin{array}{c} \text{FMD}\%=\frac{{\text{DD}}_{\text{after cuff deflation}}-{\text{DD}}_{\text{baseline}}}{{\text{DD}}_{\text{baseline}}}\times 100. \end{array}$$

In addition, Doppler signals were performed to acquire blood flow velocity in baseline conditions and at specific moments during the RH period. Doppler signals were used to obtain the brachial* shear rate* (SR) and its percentage of change, relating mean blood flow velocity (*V*_*m*_ [cm/s]) to brachial mean diameter (*D*_*m*_) according to the following equations:

(4) $$\begin{array}{c} \text{SR}=\frac{V_{m}}{D_{m}},\\ \text{SR}\%=\frac{{\text{SR}}_{\text{after cuff deflation}}-{\text{SR}}_{\text{baseline}}}{{\text{SR}}_{\text{baseline}}}\times 100. \end{array}$$

SR is an estimate of* shear stress* without accounting for blood viscosity [30] and was obtained for the characterization of the endothelial stimulus. All measurements were done by the same trained operator. The study protocol is represented in Figure 1.

#### 2.3.2. Carotid-to-Radial Pulse Wave Velocity

Noninvasively, carotid and radial pressure waveforms were simultaneously obtained using strain gauge mechanotransducers (Motorola MPX 2050, Motorola Inc., Corporate 1303 E. Algonquin Road, Schaumburg, Illinois 60196, USA) by placing them on the skin over the carotid and radial sites (left hemibody). PWVcr was determined taking into account the given distance between these arterial sites (C-R  Δ*x*) and the time delay (Δ*t*) between the carotid and radial waveforms onset (Figure 1). The algorithm used for the detection of the foot waves was described and explained in previous work [31]. Although a four-minute recording postcuff release was obtained, one-minute postischemia was the specific moment where the analysis was especially taken, according to previous reports [14, 31, 32]. The PWVcr accepted variation coefficient was less than 7%.

PWVcr levels corresponding to baseline and to post-ischemia period were determined by averaging eight consecutive beats. After that, percent of change of PWVcr (with respect to basal levels) was quantified as follows:

(5) $$\begin{array}{c} \Delta \text{PWVcr}\%=\frac{{\text{PWVcr}}_{\text{after cuff deflation}}-{\text{PWVcr}}_{\text{baseline}}}{{\text{PWVcr}}_{\text{baseline}}}\times 100. \end{array}$$

### 2.4. Statistics

The statistical analyses were performed using the Statistical Package for Social Sciences (version 19.0). All data were expressed as mean value (MV) ± standard deviation (SD) and a *P* < 0.05 indicates significant statistical differences. Comparisons between pregnant and non-pregnant women were performed using two-tailed unpaired Student *t*-test. Differences in prevalence were analyzed using *χ* ^2^ Test with or without Yates's correction if appropriate. Differences in percentage of change of variables determined before and after the VRT (arterial diameter, PWV and shear rate) were evaluated using two-tailed paired Student *t*-test. Linear regression analyses were used to assess relationships between variables.

## 3. Results

Recordings were successfully obtained from all women and all studies were included in the analysis. The mean duration of the studies was one hour approximately and they were all well tolerated (without symptoms and/or complications). The mean gestational age at examination in HP women was 34.2 ± 3.3 weeks. Demographic, anthropometric, and clinical data are shown in Table 1. As was mentioned above, age, height, and cardiovascular risk factors were taken into account in order to match groups. Body mass index was significantly higher in HP compared with NP (*P* < 0.05).

Baseline cardiovascular characteristics are given and compared in Table 2. The mean heart rate was higher in pregnancy compared to the nonpregnant controls. In addition, baseline pDBP and MBP levels were significantly higher in NP in comparison with HP (*P* < 0.05). No pSBP differences were found between the groups. However, in addition to differences in cDBP, significantly higher values in cSBP were evidenced in NP women.

Mean AIx@75 was higher in NP with respect to HP (18.8 ± 10.1% versus 8.9 ± 8.6%; resp.; *P* = 0.019), whereas amplification ratio was significantly reduced (Table 2).

When analyzing geometrical and biomechanical characteristics of muscular peripheral arteries important differences were found between groups. In concrete, basal brachial SD and DD were significantly increased in HP versus NP (*P* < 0.001) and PWVcr values were higher in NP compared with HP (*P* = 0.003). Elastic arteries such as aorta and CCAs were also analyzed by both regional and local arterial stiffness parameters (Table 2). Meaningfully differences in stiffness were only evidenced in regional stiffness (PWVcf), which was significantly reduced in HP. Values of local stiffness (*E*_*P*_) of both right and left CCA did not reach statistical differences among groups, despite the lower mean values that were found in HP.

None of the groups presented atherosclerotic plaques. Structural differences were only noticed on the right CCA, where CIMT was significantly reduced in HP with respect to NP women (*P* = 0.044).

Taking into account the VRT, all groups evoked endothelial stimulus (reactive hyperemia) evaluated by changes in SR before and after cuff deflation (*P* < 0.001) (Table 3). Pregnancy was associated with increased baseline SR values in comparison with NP controls (66.2 ± 24.4 s^−1^ versus 110.8 ± 40.1 s^−1^; *P* < 0.001). Nevertheless, after cuff deflation, peak SR and ΔSR% were not different between groups (*P* = 0.079 and *P* = 0.525, resp.), ensuring a similar hyperemic stimulus (Table 3). As was expected, no significant changes were found in heart rate or blood pressure intra- and intergroup before and after the cuff deflation (data not shown).

Regarding the FMD, both groups showed dilatation of the brachial artery with respect to basal state, but as was expected, HP women showed quantitatively the highest FMD response (9.6 ± 3.4% versus 7.1 ± 2.3%; *P* < 0.05) (Table 3), despite the higher basal brachial artery diameter existing in HP (Table 2). One minute after cuff deflation, PWVcr significantly decreased in both HP (6.8 ± 1.4 to 5.8 ± 0.9 m/s; *P* = 0.005) and NP (8.4 ± 1.1 to 7.4 ± 0.9 m/s; *P* < 0.001). The mean absolute change of the study groups was similar; however PWVcr response in % differed comparing HP women with NP women (−14.0% versus −8.5%; *P* < 0.05) (Table 3). L-FMC of the brachial artery occurred in both groups independently of the physiological status (*P* < 0.001). However, even though maximal vasoconstriction of the brachial artery (negative values) was observed in HP women (−7.0 ± 4.7%; *P* < 0.001) in comparison to NP (−5.7 ± 2.4%; *P* < 0.001), the magnitude of the reduction of arterial diameters was not statistically different between the groups (Table 3).

Finally, there were no significant correlations between endothelial function parameters (FMD, L-FMC, or ΔPWVcr%) and AIx@75, PWVcf, or central blood pressure (data not shown). However, FMD correlated with L-FMC (*R* = 0.54, *P* = 0.038) and ΔPWVcr% (*R* = 0.419, *P* = 0.037), whereas no significant correlation was evidenced between ΔPWVcr% and L-FMC (*R* = 0.30, *P* = 0.198). A positive correlation between L-FMC (negative number) and basal SR (positive number) was found across the whole studied population (*R* = 0.587, *P* = 0.017).

## 4. Discussion

The present study is, to our knowledge, the first one to determine and assess simultaneously, in a group of healthy pregnant women, the endothelial function by using three different but complementary methods in conjunction with the determination of central and peripheral structural and functional arterial validated parameters. These approaches allow us to conclude that, with respect to nonpregnant women matched by age, anthropometric features and cardiovascular risk factors, pregnant women showed (1) reduced aortic and “upper limb” arterial stiffness levels, in coherence with the higher basal brachial artery diameters that were found in this group; (2) reduced central (aortic), but not peripheral, systolic blood pressure, determined by a reduced contribution of reflected waves to central aortic pressure waveform (lower AIx@75); (3) an enhanced recruitable (FMD), but not resting (L-FMC), endothelial function, despite higher basal brachial diameters.

### 4.1. Physiological Considerations

Regarding the hemodynamic parameters, we found that HP women showed, in comparison to controls, increased HR and reduced pDBP and MBP in basal state. These findings are in consonance with an expected pregnancy-induced decrease in peripheral vascular resistance and increased cardiac output at rest. However, pregnancy-related changes were also notable when central hemodynamics is analyzed. Central (aortic) systolic blood pressure and PWVcf (aortic stiffness) were lower in HP comparing to controls, independently of brachial systolic blood pressure levels. In addition, AIx@75, a composite parameter of systemic arterial stiffness and wave reflection amplitude, was different between HP and NP. This suggests that healthy pregnancy is associated with reduced wave reflection contribution to the central aortic pressure waveform and central arterial (aortic) stiffness. However, changes in CCA stiffness (*E*_*p*_) related with pregnancy were not statistically significant, despite lower mean levels in HP. This finding supports Kärkkäinen et al. report, since these authors evidenced that carotid arterial distensibility (inverse of *E*_*P*_) decreased towards the end of the pregnancy reaching the lowest values in the third trimester [27]. Nevertheless, taking our results together, we evidenced that healthy pregnancy is associated with reduced aortic stiffness, central systolic pressure, and wave reflections.

Among the methods that allow for measurement of endothelial function in the clinical setting, FMD has rapidly gained popularity because of its simplicity, reproducibility, and noninvasiveness [2, 28]. However, as was mentioned earlier, one important limitation of FMD is that it only provides information about the “recruitability” of endothelial function (i.e., its responsiveness to a specific stimulus) and not about “resting” endothelial function (i.e., release of endothelial autacoids before FMD measures are initiated) [8]. We here analyze, in healthy pregnant women, both types of functional aspects of endothelial function, “endothelial recruitability” through FMD and PWVcr changes and “resting endothelial tone” through L-FMC. As it was expected, the magnitude of FMD observed in HP in response to VRT surpassed that observed in NP. This finding, which is similar to that described in previous reports, is in coherence with an enhanced endothelial function assessed by this method [6, 7]. On another side, when analyzing changes in arterial stiffness due to VRT, even though both groups showed a reduction in PWVcr values, HP showed the major decrease (*P* < 0.05). It is noteworthy that both groups showed a similar “endothelial stimulus,” since peak SR and ΔSR% were similar between them. In addition, even though starting (basal) levels of brachial diameter and PWVcr (basal state) in HP were higher and lower, respectively, these values were not correlated with the vascular response (i.e., FMD and ΔPWVcr%). When examining the relationship between FMD and PWVcr the analysis should take into account the Moens and Korteweg equation. In that sense, PWV is determined by both arterial diameter and the elastic modulus, among other factors [12]. In a previous study, we evidenced in healthy subjects that changes in PWVcr due to the VRT may be also provoked by a smooth muscle relaxation. If there were a right shift in the brachial pressure-volume loop, post-cuff deflation, in addition to a slope decrease due to smooth muscle relaxation, the global response might suit our results (as PWVcr changes were even higher than those only expected by changes in VMF) [3]. In light of our results, we hypothesize that besides an enhanced vasodilatory response, smooth muscle relaxation could be pronounced in healthy pregnancy.

Taking into account “resting” endothelial tone, our results show that, during the cuff inflation, L-FMC occurred in the brachial artery independently of the physiological status. However, mean L-FMC was not different between the groups. Although L-FMC was firstly described at the radial artery as a specific phenomenon [9], in the 90's it had been already reported in the brachial artery in response to the cuff occlusion in subjects with hypercholesterolemia [33]. Later, Spiro et al. evidenced that this specific phenomenon also occurs in healthy subjects at the brachial artery and it can be measured reliably [34]. Studies agree that vasoconstriction of the radial artery occurs during the cuff inflation in nonpregnant women, although the mechanisms involved remain not completely understood [9, 35, 36]. L-FMC of the brachial artery has been controversial and several studies demonstrated conflicting results [33–35, 37, 38]. Indeed, Weissgerber et al. did not evidence L-FMC in the brachial artery in pregnant women [35], in contraposition with our results. Differences in cardiovascular profile, technical aspects, methodological issues, and/or interobserver variability could explain the widely variable results, as it occurs with FMD measurements [39–41]. For example, we here measure L-FMC of the brachial artery in a regimen of low but not zero blood flow (as it occurs in the radial artery) in a level that is upstream of the occlusion site. Therefore, the magnitude of reduced blood flow in the brachial artery and its relationship with the basal levels (endothelial “negative” stimulus for vasoconstriction) should surely yield different brachial responses. Nevertheless, our results indicate that the more the basal blood flow (or SR), the more the vasoconstriction of the brachial artery provoked by the occlusion of the pneumatic cuff. Even though we did not measure “the residual” brachial blood flow during the cuff occlusion, it is expected that the absolute change in brachial blood flow may be of greater amount with the same occlusion protocol, if the starting point of blood flow in basal conditions is increased (i.e., increased “negative” stimulus for vasoconstriction).

We found a significant correlation between FMD and L-FMC, FMD and ΔPWVcr%, but not between L-FMC and ΔPWVcr%. Our results indicate that brachial artery responses to inflation and deflation of the cuff related with endothelial dynamics could share some vascular mechanism. However, there are confusing results around the FMD and L-FMC correlation, with variable results depending on the analyzed artery (brachial versus radial) and type of physiological or pathophysiological circumstance [8–10, 34]. This emphasizes again the complexity of studying “endothelial functions.” Although both L-FMC and FMD are an expression of the vascular reactivity in response to changes in blood flow, their relationship is neither conceptually simple nor mathematically linear [10]. Nevertheless, it is reasonable to think that the same vasodilatatory mechanisms (i.e., nitric oxide) involved in response to increased blood flow (shear stress) will diminish (with the consequent vasoconstriction) when the stimulus for its production is reduced/abolish.

### 4.2. Interpretation of Findings

The important additional information brought by the introduction of changes in PWVcr and L-FMC, together with the information of central and peripheral hemodynamics, is that these variables provide information concerning different aspects of vascular reactivity and endothelial function, therefore complementing (and not overlapping) the information provided by FMD. An enhanced response and/or increased vasodilator reserve to changes in blood flow in a concrete vascular ledge (i.e., brachial artery) implicates an elevated capability of the arterial system to accomplish an appropriate vascular adjustment against hemodynamic changes in the long term (fetal growth) and even in the short term (exercise, change of position, etc.). In addition, these could be associated with cardiovascular benefits reported by other authors like reduced left ventricle afterload and improve diastolic function and reduced myocardial oxygen demand in the maternal circulation [42–44]. Previous results of our group suggested that pregnancy-induced hypertension (i.e., preeclampsia-eclampsia syndrome) could be associated with increased central aortic pressure, elastic arteries stiffness, and wave reflections, in conjunction with both resting and recruitable endothelial dysfunctions [45]. These arterial disturbances would not only blunt the mentioned hemodynamic benefits of the pregnancy physiological condition but also add extra load to the maternal circulation in the context of increased cardiac output and fetal requirements. However, although this comprehensive arterial assessment would improve our understanding of the haemodynamics of both healthy pregnancy and pregnancy-related diseases, the inclusion of this information together with the recognized validated clinical, obstetric, and laboratory variables remains to be addressed during the first trimester of pregnancy, since at this time they could have an additional/complementary value in the prediction of preeclampsia [46, 47]. In this small study, that addresses the feasibility of measuring these parameters simultaneously, simply, and noninvasively, we found encouraging results that, we believe, warrant further investigation.

### 4.3. Limitations and Perspectives

The sample size of our study was relatively small. However, our findings were statistically significant and, by definition, this indicates that the study was adequately statistically powered. Our technical approaches including the use of both multiple types of automated and semiautomated edge-detection/point software in ultrasound image and pressure wave assessment are largely operator independent and also empower our findings [28]. Given the means of the different variables and SDs observed in previous works and in the present sample, twenty-eight subjects (*n* = 28) of the total sample size (the sum of the sizes of comparison groups) would be required to detect a statistically significant effect of the pregnancy status with at least 80% of power [45]. Secondly, even groups were properly matched, women with dyslipidemia were included in the analysis, and this could have an impact in our results, being a limitation of the “healthy groups.”

This vascular approach may provide a more comprehensive assessment of vascular state and endothelial function in normal pregnancy. Future studies will have to determine if accounting of this information, particularly during the early stages of pregnancy (i.e., first trimester), in conjunction with recognized important clinical, obstetric, laboratory variables, will be able to improve early detection of pathophysiological circumstances like pregnancy-induced hypertension.

## 5. Conclusion

With respect to nonpregnant women matched by age, anthropometric features and cardiovascular risk factors, pregnant women showed (1) reduced aortic and “upper limb” arterial stiffness levels, in coherence with the higher basal brachial artery diameters that were found in this group; (2) reduced central (aortic), but not peripheral, systolic blood pressure, determined by a reduced contribution of reflected waves to central aortic pressure waveform; and (3) an enhanced recruitable, but not resting, endothelial function, despite higher basal brachial diameters.

## Figures and Tables

**Table 1:** Demographic, anthropometric, and clinical characteristics.

Variable	NP	HP	*P* value^*∗*^
*N*	22	11	
Age (years)	27.9 ± 6.2	29.1 ± 4.7	0.475
Gestational age (weeks)	N/A	34.2 ± 3.3	N/A
Weight (kg)	61.8 ± 7.1	66.9 ± 7.4	0.065
Height (cm)	158.6 ± 8.2	157.9 ± 7.1	0.755
BMI (kg/m^2^)	24.7 ± 2.9	27.0 ± 3.7^*∗*^	0.048
Carotid-to-radial distance (cm)	60.2 ± 9.1	61.6 ± 4.2	0.550
Carotid-to-femoral distance (cm)	59.0 ± 2.6	58.5 ± 3.2	0.665
SSN-to-carotid distance (cm)	8.0 ± 1.1	7.6 ± 0.8	0.262
Hypertension (%)	0.0	0.0	N/A
Dyslipidemia (%)	20.0	18.1	0.700
Diabetes (%)	0.0	0.0	N/A
Cardiovascular disease (%)	0.0	0.0	N/A

Values are expressed as means ± SD or as prevalence in %. *∗* indicates *P* < 0.05. All comparisons were determined using two-tailed unpaired student *t*-test and *χ* ^2^ Test with or without Yates's correction if appropriate. *n*: number of patients per group; N/A: not applicable; NP: nonpregnant women; HP: healthy pregnant women; BMI: body mass index; SSN: suprasternal notch.

**Table 2:** Central (aortic) and peripheral arterial structural and functional parameters.

Variable	NP	HP	*P* value^*∗*^
Heart rate (beats/minute)	73.8 ± 10.8	85.0 ± 10.0^*∗*^	0.007
Peripheral SBP (mmHg)	113.0 ± 11.2	112.0 ± 9.2	0.740
Peripheral DBP (mmHg)	72.4 ± 8.2	62.5 ± 8.9^*∗*^	0.004
MBP (mmHg)	89.0 ± 8.5	78.6 ± 5.7^*∗*^	0.002
Peripheral PP (mmHg)	45.9 ± 9.3	49.8 ± 17.8	0.235
Central SBP (mmHg)	104.2 ± 9.9	95.8 ± 6.8^*∗*^	0.043
Central DBP (mmHg)	72.2 ± 8.2	63.4 ± 9.4^*∗*^	0.016
Central PP (mmHg)	32.0 ± 4.3	32.4 ± 11.0	0.683
Amplification ratio (pPP/cPP)	1.31 ± 0.21	1.51 ± 0.16^*∗*^	0.006
AIx@75 (%)	18.8 ± 10.1	8.9 ± 8.6^*∗*^	0.019

Carotid-to-radial PWV (m/s)	8.4 ± 1.1	6.8 ± 1.4^*∗*^	0.003
Brachial SD (mm)	3.1 ± 0.3	3.9 ± 0.3^*∗*^	<0.001
Brachial DD (mm)	2.9 ± 0.3	3.7 ± 0.3^*∗*^	<0.001

Carotid-to-femoral PWV (m/s)	8.1 ± 1.3	6.8 ± 0.9^*∗*^	0.022

Right CCA SD (mm)	6.8 ± 0.6	7.0 ± 0.5	0.311
Right CCA DD (mm)	6.7 ± 0.5	6.9 ± 0.7	0.388
Right CCA *E*_*P*_ (mmHg)	416.9 ± 157.6	350.6 ± 110.0	0.301
Right CIMT (mm)	0.550 ± 0.077	0.462 ± 0.089^*∗*^	0.044

Left CCA SD (mm)	6.6 ± 0.6	7.2 ± 0.4^*∗*^	0.040
Left CCA DD (mm)	6.0 ± 0.5	6.5 ± 0.3	0.062
Left CCA *E*_*P*_ (mmHg)	401.8 ± 155.7	352.7 ± 179.0	0.702
Left CIMT (mm)	0.572 ± 0.090	0.519 ± 0.080	0.146

Values are expressed as means ± SD. *∗* indicates *P* < 0.05. All comparisons were determined using two-tailed unpaired Student *t*-test. NP: nonpregnant women; HP: healthy pregnant women; SBP, DBP, MBP, and PP: systolic, diastolic, mean, and pulse pressure, respectively. pPP and cPP: peripheral and central pulse pressure, respectively. AIx@75: augmentation index adjusted to a heart rate of 75 beats/minute; PWV: pulse wave velocity; SD and DD: systolic and diastolic diameter, respectively; *E*_*P*_: Peterson's or pressure-strain elastic modulus; CCA: common carotid artery; CIMT: carotid intima-media thickness.

**Table 3:** Vascular reactivity parameters: endothelial function.

Variable	NP	HP	*P* value^*∗*^
Basal SR (s^−1^)	66.2 ± 24.4	110.8 ± 40.1^*∗*^	<0.001
Peak SR (s^−1^)	180.0 ± 73.7	227.0 ± 67.8	0.079
ΔSR (%)	132.5 ± 59.9	110.9 ± 88.5	0.525

FMD (%)	7.1 ± 2.3	9.6 ± 3.4^*∗*^	0.039
ΔPWVcr (%)	−8.5 ± 6.4	−14.0 ± 7.8^*∗*^	0.035
L-FMC (%)	−5.7 ± 2.4	−7.0 ± 4.7	0.208

Values are expressed as means ± SD. *∗* indicates *P* < 0.05. All comparisons were determined using two-tailed unpaired Student *t*-test. NP: nonpregnant women; HP: healthy pregnant women; FMD: flow-mediated dilation; L-FMC: low-flow-mediated constriction; PWVcr: carotid-to-radial pulse wave velocity; SR: shear rate.
